# A Comprehensive Review of the Biochar-Mediated Alleviation of Salt Stress

**DOI:** 10.3390/plants15111699

**Published:** 2026-05-30

**Authors:** Murugesan Chandrasekaran, Iro Kang, Sivasankaran Ayyaru, Jagadeesh Kumar Alagarasan, Iyaakannu Sivanesan

**Affiliations:** 1 Department of Food Science and Biotechnology, Sejong University, 209 Neundong-ro, Gwangjin-gu, Seoul 05006, Republic of Korea; chandrubdubio@sejong.ac.kr; 2 Department of Plant and Soil Sciences, College of Agriculture and Natural Resources, University of Delaware, 531 South College Avenue, Newark, DE 19716, USA; irokang@udel.edu; 3 Department of Bioengineering, Saveetha School of Engineering, Saveetha Institute of Medical and Technical Sciences, Chennai 602105, Tamil Nadu, India; sivasankaran2010@gmail.com; 4 Department of Civil Engineering, Yeungnam University, Gyeongsan 38541, Republic of Korea; 5 Centre for Research Impact and Outcome, Chitkara University, Rajpur 140401, Punjab, India; 6 Department of Chemistry, Manipal University, Jaipur, Dhmikalan 303007, Rajasthan, India; jaga.jagadeesh1987@gmail.com; 7 Department of Environmental Health Science, Human and Eco Care Center, Konkuk University, Hwayang-dong, Gwangjin-gu, Seoul 05029, Republic of Korea

**Keywords:** biochar, modified biochar, salt stress, stress resilience

## Abstract

Salt stress remains a major global stress factor among abiotic stresses limiting crop production. Salt stress is a major nutritional challenge, with poor agricultural production characterized by high soil sodium (Na^+^) levels in soil and plants. Soil salinity negatively affects plants through both osmotic effects and ionic toxicity. Hence, one of the main aims of agricultural scientists is to develop eco-friendly, sustainable solutions to alleviate soil salinity. Over the past decades, several studies have recommended biochar as a vital sustainable soil amendment to alleviate the negative consequences of soil salinity. Thus, this review builds on the literature on biochar-mediated alleviation of salt stress. Biochar is a carbon-rich material produced from biomass and feedstock via pyrolysis under little or no oxygen conditions. Due to its unique characteristics, such as high carbon, high surface area with porous and aromatic structure, high pH, high stability, cation exchange capacity, and water and nutrient retention capacity, it is considered an alternative for salt stress alleviation. Moreover, biochar facilitates sodium ion (Na^+^) adsorption, reduces Na^+^ uptake, and increases potassium ion (K^+^) uptake, enhancing nutrient cycling, helping plants maintain ionic balance and osmotic regulation. This, in turn, significantly increased the activity and diversity of soil microorganisms, enhanced their adhesion, and promoted their growth, thereby strengthening the plant’s salt resistance. Moreover, biochar-mediated improvements in microbial community dynamics and changes in the physical and biological properties of soil contribute to overall plant and soil health under salt stress. Hence, the present review aims to decipher the holistic patterns of biochar on soil and plant health, changes in physiological and defense mechanisms, plant hormones and signaling mechanisms, and the status of modified biochar under salt stress. Thus, the present review will pave the way for the production of salt-resilient crops with enhanced salinity tolerance. In conclusion, the use of biochar-based fertilizers and modified biochar enhanced microbial community dynamics in soil health homeostasis and soil fertility for agricultural production and food security.

## 1. Introduction

Biochar is a porous carbon-rich solid material produced from biomass and other agricultural waste in the absence of oxygen (or little oxygen). Biochar is generally synthesized by pyrolysis through a thermal process at temperatures (250–900 °C) under limited O_2_ conditions [1,2]. Different types of biomass and thermochemical conditions greatly influence the quality and type of biochar, as well as its potential uses. Several studies showed that biochar had a high carbon content, large surface area, ion exchange capacity, low bulk density, and high water-holding capacity [1,3,4]. Moreover, biochar changes the physical, chemical, and biological characteristics of soil. Recent studies suggested that biochar is a vital soil amendment for soil fertility, nutrient cycling, and carbon sequestration; soil–plant–microbe interactions; an adsorbent for the removal of toxins from wastewater and polluted soils, as well as heavy metals and pollutants; and acts as a habitat for soil-beneficial microbes that increase soil fertility [1,3,4,5]. Due to its beneficial characteristics, biochar is used as a soil amendment for agricultural applications and food security.

The world population is expected to reach 10 billion by 2050 (FAO, 2025), putting a considerable amount of pressure on crop production to meet ever-increasing food demand. Frequent exposure of agricultural crops to abiotic stresses (drought, salinity, and heavy metals) due to climate change, land degradation, and environmental pollution results in significant yield loss and reduced plant productivity [5,6]. Among abiotic stresses, soil salinization and its effects on crops have emerged as a critical threat to agricultural production. Soil salinity is aggravated by both natural causes and anthropogenic activities such as urbanization and deforestation. Further, the cumulative effects of low precipitation, poor drainage and irrigation, and high evaporation influence crop production (depending on environmental stress and the ever-increasing population) [6,7]. Previous reports indicate that nearly 800 × 10^6^ million ha of land are affected by salt stress, with an annual increase of 1–2% aggravating the impact globally [7,8]. Salt stress induces land degradation, resulting in an annual economic loss of USD 27.2 billion from irrigation and subsequent crop losses [8]. Sustainable crop production relies on effective interactions between soil moisture and salinity [9]. Therefore, salt stress poses a major constraint on global food security.

Soil salinity causes ionic imbalances and osmotic stress in plants, impairing their water and nutrient uptake. Osmotic stress quickly affects plants, leading to higher salt levels in the soil solution around roots and reduced water uptake, whereas ionic stress involves excessive accumulation of toxic ions (Na^+^) beyond threshold levels, which minimizes cellular metabolism, leading to increased leaf mortality, chlorosis, necrosis, and decreased photosynthesis. This change in Na^+^ levels causes stunted growth and lower yields. Additionally, nutrient transport from roots to shoots is disrupted, causing nutrient imbalances that adversely affect various physiological and biochemical processes in plants [10]. Moreover, increased accumulation of sodium ions (Na^+^) in plant tissues negatively affects essential metabolic processes. Soil salinity causes several negative effects on plant physiology, including reduced leaf water content, growth, yield, nutrient uptake, and breakdown of the photosynthetic machinery [11,12]. Moreover, oxidative stress in plants increases ROS production to combat salt stress. Elevated reactive oxygen species (ROS) levels in plants create an imbalance between oxidative production and antioxidant defense mechanisms. It leads to lipid peroxidation of cellular membranes and accumulation of high levels of malondialdehyde (MDA), leading to severe cellular damage. High salinity in the root zone prevents optimum yield and plant growth [10,11,12,13]. Soil salinity increases the concentration of Na^+^ and Cl^−^ in soil solution, which in turn accumulates in soil and is also translocated to the above parts of the plants. It leads to soil degradation, nutrient imbalances, impaired hydraulic activities, and limited water availability, which in turn clearly limit agricultural production in saline soil [14,15,16,17]. Various investigations indicate that higher salinity is the primary cause of reduced crop production and yield loss compared to other environmental stresses [16,17,18]. Hence, alleviating salt stress and mitigating soil salinization are foreseen as crucial challenges worldwide. Hence, dire attention is focused globally on combating salt stress and enhancing agricultural production.

Recent attention to the mitigation of soil salinization has been focused on nature-based solutions and modern bioengineering in the breeding and production of salt-resilient crops [1,7]. Amelioration of soil salinity is mainly achieved through the application of organic matter. Biochar is a carbon-rich, charcoal-like organic material derived from waste materials such as poultry manure and animal or crop residues [18]. Biochar production relies on exothermic processes and pyrolysis (in the absence of or with low oxygen at high temperatures), improving plant growth [2,19]. Biochar-mediated plant growth promotion under salt stress occurs by two mechanisms. The former corresponds to direct action by supplementing plant mineral nutrients (calcium (Ca), K, sulfur (S), phosphorus (P), and Mg), while the latter indirect mechanism enhances soil physicochemical and biological properties contributing to plant growth [7,11,20]. Alterations in physicochemical properties include water-holding capacity, pH, CEC (cation exchange capacity), soil structure, and surface area. Biological processes augmenting plant growth include improved germination percentage. Moreover, the recalcitrant properties of biochar are attributed to higher aromaticity, ash content, pH, nutrients, and total carbon, which are improved by pyrolysis temperature and the matter utilized during biochar production [21]. Variability in low-to-high temperatures influences the pH range during pyrolysis, leading to either alkaline or acidic conditions. Acidic biochar corresponds to higher pH, alkalinity (calcium carbonate, CaCO_3_), and electrical conductivity (EC), resulting in higher soil pH and EC. Previous studies have shown that using alkaline biochar (pH 7.0) can increase soil EC and pH compared to acidic biochar [22,23].

Biochar application is also used as a soil amendment for salt exclusion, salt sequestration and associated mechanisms, and improving soil physical, chemical, and biological properties [20,24,25,26]. Further, in plants, biochar mitigates salinity stress by improving K^+^ uptake and reducing Na^+^ uptake, thereby enhancing the gas exchange and water status of plants [27,28]. Previous studies also demonstrated that applying biochar increased the concentrations of soil-available K, P, and organic matter, which in turn reduced toxic ions (sulfate (SO_4_^2−^), Na^+^, and chloride (Cl^−^) levels) in soil [20,22,24,25]. The use of biochar is regarded as a mutually beneficial approach for attaining numerous benefits within the realm of sustainable agriculture. Hence, the present review aims to elucidate the holistic patterns of ionic exclusion/inclusion, homeostasis, K^+^ uptake, and enhanced physiological outcomes. This review will pave the way for the development of salt-resilient crops with enhanced salinity tolerance. Further, this review emphasized the need for additional insights into the role of biochar in soil microbial community dynamics, soil health, plant physiological and biochemical mechanisms, and changes in phytohormones, antioxidants, and plant signaling cascades under salt stress conditions. Nanotechnology-based modification of biochar is illustrated for future directions. Nevertheless, challenges and limitations regarding the potential agronomic and environmental risks associated with biochar treatment are delineated for novel applications.

## 2. Biochar Production

**Figure 1:**
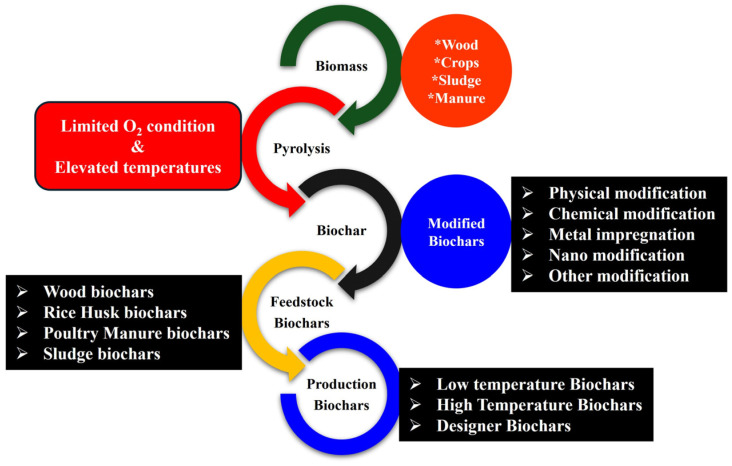
Different methods of Biochar production.

Biochar baseline production depends on thermochemical combustion of organic materials under limited oxygen supply (Figure 1). Prehistoric biochar production dates to charcoal preparation on Earth and the aboveground pile combustion of large wood stocks. The type of feedstock determines biochar quality and its ability to deliver multiple benefits for sustainable agriculture [2,19,29]. Common methods of biochar production include rotating, vertical silo-type reactors, gasification, hydrothermal carbonization, and pyrolysis (slow and fast) [30]. A high concentration of total organic carbon (30–70%) depends on other pyrolysis conditions (temperature and time), high mineral contents (Na, K, Mg, iron (Fe), etc.), high pH and EC, and low ash and volatile matter [30,31] (Figure 1).

Further, sophisticated techniques are used for characterization, quantification, and identification of biochar and its surface functional groups, aromatic compounds, polycyclic aromatic hydrocarbons, and active surface area [30,31,32,33]. Furthermore, the types of feedstock and production techniques determine the quality and efficiency of biochar. For example, the adsorption capacity resulting from surface functional group modification during pyrolysis depends on the feedstock and temperature. The pyrolysis mechanism involves the loss of easily degradable organic feedstock compounds (cellulose and lignin) at 300 °C and above, which leads to the condensation of aromatic compounds and an increase in surface area [34,35,36]. Hence, several modifications have been reported for enhanced functional properties. Four biochars differing in feedstock (wood, bamboo, rice husks, and rice husk ash) showed more carboxyl, lactone, and phenolic groups in wood biochar than in the others [37]. High aromaticity was observed in wood biochar (charcoal) compared with sewage sludge derived from hydrothermal carbonization coal (HTC) and low-temperature conversion coal (LTC) [38]. Moreover, the functional groups of pine-needle-derived biochar, as determined by Boehm titrations, depicted key variations. Removal of acidic functional groups (carboxylic, phenolic, and lactones) with accumulation of basic functional groups (ketones, pyrones, and chrome) [39] established corporation potential in soils and water for economic removal of contaminants and pollutants [40,41,42].

Previous studies showed that application of biochar can be used for augmentation of chemical, physical, and biological properties of soil [43], carbon dioxide (CO_2_) sequestration [44], removal of heavy metals [45,46], as an active metal catalyst [47], and for reducing greenhouse gas (GHG) emissions [48]. Substrates for biochar production include agricultural waste, forest waste, bio-based industrial waste, municipal soil waste, sewage sludge, animal manure, and aquatic biomass. Several methods, such as pyrolysis (fast, slow, and flash, vacuum, and microwave), hydrothermal carbonization (HTC), flash carbonization, and gasification have been reported for augmented biochar properties [49]. Algal biochar benefits with soil amendment and pollutant (organic and inorganic) removal [50]. Moreover, pristine and modified biochar played a major role in the removal of micro (nano) plastics [51].

Biochar production, which impacts soil health, crop yield, and productivity, is grouped under thermal conversion (roasting, gasification, torrefaction, flash carbonization, and hydrothermal carbonization). Hence, effective impacts include enhanced physical properties (bulk density, soil compaction, porosity, water-holding capacity), soil chemical properties (pH, salinity, sodicity, cation exchange capacity, nutrient availability and retention), and biological properties. Further effects include enhanced yield and regenerative agriculture exploring diverse outcomes (soil fertility, carbon sequestration, microbial community dynamics, remediation, water quality, and enhanced resilience) [52]. Moreover, nanotechnology-modified biochar mineral fertilizer (BMF) is effective in long-term climate resilience [53]. Hence, a separate section on biochar-based biofertilizers and nanotechnology-modified biochar is fostered to collate soil and plant needs. However, the cumulative aspects of biochar-mediated salt stress alleviation are addressed by examining intricate soil mechanisms that augment cellular, biochemical, and physiological outcomes in plants. Furthermore, soil microbial community dynamics, phytohormones, antioxidant defense enzymes, non-enzymatic alleviation, and plant signaling mechanisms are summarized.

## 3. Biochar Effects on Soil and Plant Health

### 3.1. Soil Microbial Community Dynamics

Soil is home to numerous microorganisms; they play a key role in soil properties and processes. Many studies have shown that salt-stressed soil reduces microbial activity and biomass and alters microbial community structure. Furthermore, soil–root interactions vary with soil environmental conditions, playing an essential role in nutrient uptake. Adding biochar to soil changed the ion balance, affecting salt ions and pH levels, which depend on soil type, plant, and stress [54,55,56]. Several studies showed that the application of biochar in saline soil increased the content of soil organic matter. Because increased soil organic matter increases plant productivity due to changes in the porosity of the soil, there are changes in available nutrients in the soil. This significantly boosted microorganism activity and diversity improved their adhesion and supported the growth of soil microbes. As a result, it enhanced biological salt resistance and soil purification.

With the amendment of biochar, soil microbiota (bacteria, fungi, and archaea) becomes enriched, which alleviates stress and enhances plant health [57]. *Rhizobium* or the root microbiome under biochar treatment provides multiple benefits for plant resilience (nutrient acquisition, phytopathogen management, and stress management) [58]. Nutrient acquisition involves N_2_ fixation, P solubilization, and chelation of Fe [59]. Moreover, high P availability enhances the soil bacterial growth of *Plavobacterium*, *Pseudomonas*, and *Thiobacillus* for effective solubilization [60,61]. Wang et al. [62] observed that applying biochar increased the relative abundance of *Bacillus* and *Nitrospira*. Additionally, microbial richness, particularly bacterial diversity, is crucial for the changes in soil properties induced by biochar. Liu et al. [63] demonstrated that biochar application increased soil total phosphorus, thereby boosting the relative abundance and distribution of phosphate-solubilizing bacteria, including *Pseudomonas*, *Flavobacterium*, and *Thiobacillus*. Another study indicated that biochar also enriched the soil with *Proteobacteria*, *Chloroflexi*, and *Acidobacteria* [64]. Therefore, understanding the character of the soil microbial community is essential for assessing soil quality and fertility, as well as crop productivity. The more complex the soil microbial community, the more resilient the soil ecosystem tends to be.

Biochar–AMF interactions aid in carbon sequestration and enhanced water availability in temperate soils [65]. Earlier physicochemical alterations by biochar also showed reduced soil compaction, optimized compost use [66], and bio-stimulation of rhizosphere microbes and AMF benefits [67]. Biochar obtained from *Broussonetia papyrifera* leaf litter (2%) revealed enhanced enzymes (ureases, proteases, dehydrogenases, and phosphatases), depicting improved microbial function [68]. Other mechanisms are also attributed to increased hydraulic conductivity, increased soil moisture, and nutrients (K+, Ca^2+^, and Mg^2+^) foreseen for biochar–soil–plant interplay during salt stress. However, complex interactions between soil microbiome community structure dynamics and biochar inputs altering salt stress remain surprising [69]. Further, biochar has been shown to neutralize soil acidity and alleviate stress through immobilization of contaminants [20]. Detoxification of polycyclic aromatic hydrocarbons (PAHs) by biochar in soil enhanced the microbial population in soils [70]. Further, mitigating nitrous oxide and methane emissions is a significant activity of biochar in addition to the supply of macro- and micronutrients [71]. Hence, rigorous multi-omics and big data analysis will yield significant insights into deciphering key genes and transcription factors. Thus, the multi-faceted biochar–soil plant interactions are summarized. However, the ionic balances and mechanistic insights will illustrate a comprehensive positive outcome.

### 3.2. Biochar-Mediated Changes in Physiological Mechanisms

**Figure 2:**
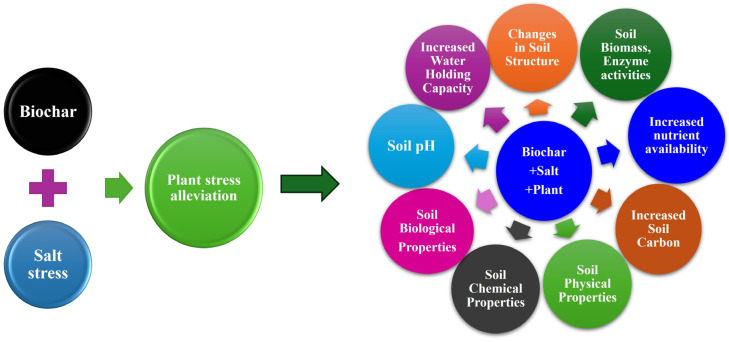
Biochar-mediated changes in soil characteristics under salt stress.

Increased soil salinity causes physiological stress in plants by disrupting ionic and osmotic balance, driven by salt accumulation in the soil rhizosphere. Salts adversely affect plants through both osmotic effects and ionic toxicity. Several agricultural studies have assessed biochar’s ability to improve biomass and crop yields. Furthermore, biochar’s ability to enhance water availability, ion exchange capacity, and nutrient retention may partly explain its effectiveness in mitigating salt stress. Thus, interactions between roots and biochar in soil may influence root function [72], potentially enhancing plant development through biochar incorporation [73,74,75] (Figure 2). In soils enriched with biochar, roots experience reduced sensitivity to osmotic pressure as moisture levels rise due to improved soil characteristics and the binding of sodium to biochar surfaces [76,77]. The introduction of biochar has been shown to increase shoot weight in maize [78] and increase the biomass of both aboveground and root structures in wheat plants [76], especially under saline stress.

Parkash and Singh [79] reported that hardwood and softwood biochar alleviates salt stress in eggplants. They found that biochar-inoculated plants increased the root length density (15%), fruit yield (18%), photosynthesis (20%), and stomatal conductance (25) under salinity than those of non-biochar-inoculated plants. In another study, acidic biochar (45 g/kg) increased maize height (25%), leaf area (30%), and biomass (35%) in saline soil, demonstrating its potential to alleviate salt stress [22]. Murtaza et al. [80] reported that the addition of *Acacia* biochar (40 g/kg) to saline soil enhanced corn biomass and *Acacia* biochar played an important role in the alleviation of soil salinity. Nevertheless, incorporating higher amounts of *Acacia* biochar (40 g/kg) significantly improved corn photosynthetic rate [80]. Abbas et al. [81] showed that cotton shell biochar (2% (*w*/*w*)) inoculated quinoa plants grown on saline–sodic soil alleviated Na toxicity by increasing biomass (60%), grain yield (18%), chlorophyll content (43%), and stomatal conductance (41%). Additionally, biochar enhances leaf photosynthetic performance and reduces oxidative stress, thereby increasing biomass and plant yield under salinity conditions [82]. Biochar treatment increases chlorophyll levels, improves photosystem II (PSII) activity, and facilitates electron transfer [83], and maximizes the content of pigments (chlorophyll and carotenoids), amino acids, and proteins in plants [84].

In addition, cow bone biochar (produced from pyrolysis of cow bones) has been used to increase plant growth. Application of cow bone biochar enhanced tomato yield, quality, and shelf life by altering soil chemical properties, as evidenced by increased total acidity, total soluble solids, and vitamin C and lycopene levels [85]. The addition of biochar positively influences wheat growth under salt stress by improving membrane stability, nutrient uptake, and chlorophyll synthesis and by altering stress-response gene expression. These improvements lead to increased productivity and greater plant resilience in challenging conditions [86]. The physico-biochemical effects of biochar in *Thymus vulgaris* under salt stress showed high porosity, water retention, reduced sodium uptake, high essential oil, volatile components such as thymol and carvacrol, and rosmarinic acid; consequently, eco-friendliness and plant soil performance were positive [87]. Peanut shell biochar and fermented cow manure, with enhanced plant metabolism, affirmed the circular economy and the effective use of agricultural wastes as feedstock [88]. However, the ionic balances and mechanistic insights will illustrate a comprehensive positive outcome.

**Figure 3:**
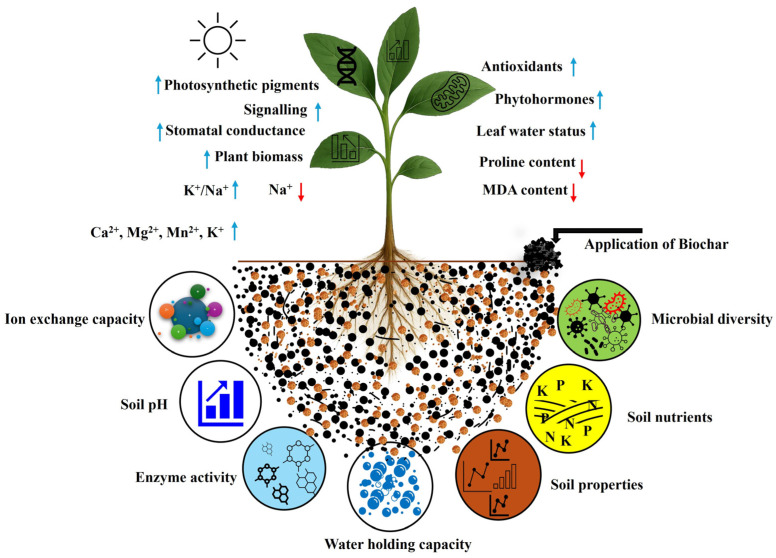
Changes in plant and soil physiological characteristics in biochar-mediated salt stress alleviation. Blue arrow ↑ indicates increased content, and red arrow ↓ indicates decreased content.

Biochar alleviates salinity stress by increasing K^+^ uptake and reducing Na^+^ uptake [84] and upregulating aquaporin genes [85], hereby enhancing plant water relations and leaf water status against osmotic stress [86]. Moreover, biochar-induced Na^+^ reduction and Cl^−^ uptake are corroborated with soil fertility, root growth, chlorophyll synthesis, stomatal conductance, osmolytes, phytohormones, antioxidants, membrane stability [24], and photosynthetic efficiency [74,75,76,77,78]. Other positive effects of biochar are attributed to increased Mg^2+^ uptake for chlorophyll synthesis [87,88], NADP reduction for increased NADPH [89], and subsequent enhancement of photosynthetic efficiency (improved hill reaction activity, PS-II integrity, electron transport efficiency, and decreased ROS) [90]. Increased uptake of P, Mn, K, Fe, and Zn counters salinity and toxicity in a dose-dependent manner, emphasizing plant performance [91,92]. The incumbent modifications were enhanced photosynthetic pigments (total chlorophyll, chlorophyll a and b) and increased N, P, K, and biomass under salt stress (Figure 3). The above impacts were correlated to soil properties and improved nutrient availability replacing the use of chemical fertilizers [93]. Other mechanisms are also attributed to increased hydraulic conductivity, increased soil moisture, and increased nutrient availability (K+, Ca^2+^, and Mg^2+^) foreseen for the biochar–soil–plant interplay during salt stress. Hence, the impact of biochar on cellular metabolism and its effect on plant health and subsequently upon soil health is critically addressed. The basic mechanism by which biochar augments agronomic traits in plants at the cellular level and overall soil health is correlated with enhanced plant growth promotion and soil fertility, thereby alleviating the need for conventional chemical fertilizers. Holistically, soil–plant–biochar effects are correlated to complex plant mechanisms (resilience, protection, nutrition, growth, system change, biological change, chemical change, and structural change) confronting dynamic and multi-dimensional interplay. Therefore, physico-biochemical biological effects induced by biochar–soil–plant interactions are rationalized. So, the multi-faceted biochar–soil plant interactions are summarized.

### 3.3. Biochar-Mediated Changes in Defense Mechanisms

Salt stress in plants disrupts ionic balance in cells, induces excessive ROS production, and causes oxidative damage to cellular organelles and biological macromolecules, leading to reduced plant growth and yield. Increased salinity causes the accumulation of MDA (an indicator of ROS) and induces lipid peroxidation. Accumulation of more MDA in cells causes severe damage to the cellular membrane, which eventually leads to leakage of electrolytes and osmolytes, resulting in K deficiency [94,95]. To combat oxidative damage caused by salt stress, plants developed strong defensive systems, including enzymatic and non-enzymatic activities and the production of osmolytes. Antioxidant enzymes such as catalase (CAT), peroxidase (POD), and superoxide dismutase (SOD) play a crucial role in the removal of ROS [95]. Several studies have shown that biochar inoculation increases antioxidant activity in plants under salt stress [96,97,98,99]. However, several studies conducted on plants such as beans, tomatoes, and corn have shown that adding biochar to saline culture media can reduce antioxidant enzyme activity [80,97,98]. Biochar increased enzymatic antioxidants and reduced ROS, hydrogen peroxide (H_2_O_2_), OH^−^, and MDA content, thereby reducing osmotic stress caused by soil salinity and enhancing spinach growth and yield [100]. Similar studies on soybeans have found that adding biochar can improve salt tolerance by reducing oxidative stress [101]. Further, biochar enhances antioxidant mechanisms and osmolyte responses, which combat MDA- and H_2_O_2_-induced toxicity by preventing electrolyte leakage [89,101,102]. Cell membrane damage was reduced by using biochar, consistent with the results of Mehdizadeh et al. [77] for *Satureja khuzistanica*. Biochar application increased antioxidant enzymatic activities (ASA-glutathione GSH [90]), ascorbate peroxidase (APX), monodehydro ascorbate reductase (MDHAR), glutathione reductase (GR) [101,102,103] and other enzymes, such as CAT, POD, and SOD [104]) and non-enzymatic antioxidant activities and proline synthesis that fight osmotic stress. The application of *Acacia* biochar (40 g/kg) mitigated the adverse effects of salt-induced oxidative stress by augmenting CAT and glutathione S-transferase (GST) activity in corn plants [80]. Researchers have reported that reduced antioxidant activity could be caused by decreased Na^+^ absorption and accumulation in crops and by reduced salinity-induced harmful effects [102,103,105]. In addition, biochar treatments in saline environments prevent excessive production of ROS, H_2_O_2_, and MDA by improving redox homeostasis, thereby reducing lipid peroxidation [77,106]. Reducing H_2_O_2_ and MDA content by using biochar decreases electrolyte leakage by strengthening the membranes [107]. Ca-rich biochar can promote the production of osmotic protectants, such as soluble sugars and proline, in plants by reducing Na+ uptake, enhancing antioxidant capacity, and alleviating oxidative stress. The application of Ca-rich biochar at 2.5% reduced leaf tissue MDA content by 32%, showing enhanced salt tolerance in the plant [108]. Biochar application also increases the concentration of non-essential fatty acids, enhancing the fluidity, stability, and function of plant cell membranes under salt stress, which ultimately increases the activities of enzymatic antioxidants and prevents the loss of vital osmolytes in plants [107,109]. Hence, biochar-mediated salt stress alleviation through antioxidants contributes to food security and improved productivity.

However, non-enzymatic antioxidants and signaling mechanisms play pivotal roles in alleviating salt stress. Non-enzymatic antioxidant mechanisms involve the ascorbate–glutathione cycle, which produces monodehydroascorbate (DHA). Furthermore, biochar application enhances the concentration of osmolytes (proline, glycine, and betaine) and secondary metabolites. Proline acts as an osmoprotectant and ROS scavenger of hydroxyl radicals and singlet O^2−^ under salt stress [110]. Ascorbic acids (ASA) aid in the regeneration of enzymatic antioxidants (H_2_O_2_, hydroxyl radicals) and ROS scavenging with APX in the ascorbate–glutathione cycle [111,112]. Glutathione (GSH) yields reducing power (lipid peroxidation and hydroxy radicals) for ROS reduction, enzymatic antioxidant regeneration, and cellular redox homeostasis [113]. Thus, the non-enzymatic mode of salt stress mitigation is complemented by biochar actions. Other mechanisms include polyamines and the thioredoxin system. The former stabilizes the membrane and supports free radical scavenging, involving putrescine, spermidine, and spermine for the integration of membrane scavenging ROS. The latter corresponds to redox balance by protein repair for reduced oxidized proteins, enzyme activity, and subsequent redox homeostasis [114]. However, osmolyte–phytohormone crosstalk is stressed for advanced multi-omics studies [24]. Further, biochar application revealed enhanced metabolic profile of leaves (increased essential amino acid, sucrose, and organic acid metabolism) in sugarcane with root growth promotion and microbial health in soil [115,116]. Biochar supplementation altered the contents of secondary metabolites in *Melissa officinalis*, thereby promoting its growth and alleviating salt stress [117]. Hence, the cumulative antioxidant and non-enzymatic antioxidants are collated to biochar-mediated salt stress tolerance.

### 3.4. Biochar-Mediated Changes in Phytohormones

Phytohormones act as important signaling molecules for crosstalk between hormones for plant growth and regulation of signaling mechanisms. Both growth- and stress-related hormones play important roles in plant tolerance to salt stress [118,119]. Previous studies have shown that biochar improves germination and seedling growth under salt-stressed environments by regulating plant hormones such as gibberellins (GAs) and cytokinins (CKs) [120,121]. GAs are associated with root growth, reduced Na+ accumulation, and root and shoot elongation [122]. CKs enhance shoot growth and chlorophyll contents and improve nutrient homeostasis [123,124]. Biochar also increases the synthesis of hormones such as indole-3-acetic acid (IAA), which improves nutrient absorption, growth, and the synthesis of essential amino acids such as tryptophan and glycine, thereby increasing crop resistance to salt stress [125,126,127]. Moreover, biochar treatments reduce the contents of endogenous stress hormones such as salicylic acid (SA), jasmonic acid (JA), and ABA by reducing Na^+^ absorption and accumulation in plants [119,125]. ABA plays a pivotal role through perception and response regulation under salt stress. ABA levels activate ABA-response element-binding proteins and dehydration-response element-binding proteins, regulating stress tolerance gene expression, osmotic tolerance, and ion homeostasis [128]. Biochar application decreased ABA levels [24] and increased plant production [129]. ABA interacts with JA (secondary metabolites for enhanced stress tolerance) [130] and ethylene (root architecture and leaf senescence for plant growth) [131]. ABA accounts for increased water-holding capacity, reduced transpiration, and increased ion homeostasis. SA reduces oxidative damage and increases detoxification enzymes (activation of antioxidant systems and regulation of stress-related genes [132]). BR enhances membrane stability and reduces oxidative damage and osmotic adjustment (increased antioxidant enzyme activity and osmolyte accumulation) [133]. Auxins were shown to enhance water and nutrient uptake, with improved root characteristics (lateral root formation and cell elongation) [134]. Importantly, stress hormones (ABA, ethylene, SA, and JA) and crosstalk mechanisms in salt stress need to be repurposed for prominent benefits [135]. Thus, the inherent crosstalk and interplay of phytohormones depend on signaling and stress response mechanisms.

### 3.5. Biochar-Mediated Changes in Signaling Mechanisms

The fundamental mechanism of biochar is its anticipated cellular interaction, which relies on ionic inclusion and exclusion, as well as biological metabolism. Other inherent actions involving physiological mechanics are characterized by complex interplay, contributing to the soil–plant–biochar nexus and soil microbial community dynamics. Hence, signaling mechanisms are being developed to enhance salt tolerance. In general, plant signaling in response to salt stress is accounted for primarily by osmotic and ionic imbalances due to Na^+^ influx, activation of secondary messengers, and subsequent regulation of ion channels and transporters. Salt stress perception in plants involves sensing increased levels of Na^+^ and Cl^−^ ions in the soil. Ion channels (cyclic nucleotide phosphodiesterase-CNPase) and transient receptor potential enable the detection of ionic changes [136,137], as well as high-affinity K^+^ transporters and Na^+^ and H^+^ exchanges (NH_x_). Moreover, Na^+^ and Cl^−^ influx are further perceived by cell sensors depending on concentration [138]. The major salt stress signaling pathway is the nitrogen-activated protein kinase (MAPK) cascade, which involves serine/threonine protein kinases [139].

Increased Ca^2+^ level and cell signaling improve salt tolerance. Recent research has also indicated the significance of the biochar Ca^2+^ signaling nexus in plant immunity. Priming defense genes with biochar and Ca2+ flux will open new areas of complex interactions in soil community structure dynamics and plant immunity [140]. The ROS pathway contributes to the expression of transcription factors and protein kinases (SNF1-related protein kinases 2 (SnRK2)) and Ca2+-dependent protein kinases (CDPKs) for the maintenance of cell homeostasis and the regulation of stress responses [141,142]. Ca^2+^ acts as a secondary messenger, sensed by calcium-binding proteins (calmodulin-CaM and calcium-dependent protein kinases-CDPKs) [142]. The perception and response of plants to salt stress are foreseen to help decipher the complex interplay. Thus, a research thrust to dissect the complex interplay of signaling cascades in soil-plant-biochar-enhanced salt stress management requires further experimental evidence.

## 4. Modified Biochar

### 4.1. Nanobiochar

Recently, nanoscale biochar has gained significant interest because of its unique physical and chemical properties. Its specific surface area is much greater than that of the original biochar [143]. Nanogrinding releases a high level of dominant organic matter (DOM), which promotes plant growth [144]. Furthermore, biochar-based nanocomposites are instrumental in treating environmental pollutants through processes like adsorption and electrochemical degradation. Using nano-modified biochar substantially boosts soil organic matter, organic carbon, and macro-nutrient availability (N, P, and K). Iron- and zinc-modified biochar enhances microbial biomass carbon and nitrogen, along with enzyme activities such as urease and β-glycosidase in saline soils. Treatments with nanobiochar alter microbial community structures, increasing the relative abundance of salt-tolerant bacteria such as *Proteobacteria*, *Chloroflexi*, and *Bacteroidota* under saline conditions, and modify fungal communities, raising the relative abundance of *Chytridiomycota* with ion-modified biochar [145]. Moreover, nanobiochars selectively modify the microbial community. Previous studies showed that nano biochars enriched the nitrogen cycle of related microbial species such as *Truepera*, *Chelativorans*, *Desulfomicrobium*, and *Chloropseudomonas*. It suggests that nanobiochar plays a major role in organic matter decomposition, nitrogen cycling, and transformation of nutrients [146,147]. Supplementation with nanobiochar elicited distinct responses across various bacterial and fungal species, underscoring species-specific interactions. When Fe-Mn-modified biochar was used, the bacterial groups *Firmicutes* and *Proteobacteria* exhibited prominent responses [148]. Similarly, applying Fe-Mn-Ce-modified biochar (which combines iron, manganese, and cerium with biochar) increased activity in the Gemmatimonadaceae family within Gemmatimonadetes and the Oxalobacteraceae family within *Proteobacteria*, indicating these families as dominant responders as their activity levels rose in the soil [149].

When biochar particles are at the nanoscale, their specific surface area increases, along with the number of active sites for chemical adsorption and overall reactivity, including the capacity to bind inorganic ions [150]. For instance, biochar nanoparticles contain more oxygen-containing functional groups and aliphatic chains, which confer greater stability and reactivity, enhancing their ability to adsorb anions [151,152]. As noted earlier, a smaller size allows biochar nanoparticles (NPs) to more effectively mitigate salt stress. Tao et al. [153] found that applying rice straw biochar NPs increased tomato seedling biomass (13%) and chlorophyll content (46.7%) under salt stress. These NPs inhibited Na transfer from roots to shoots. Similarly, corn straw biochar NPs helped reduce salt stress in tomatoes, though to a lesser extent than rice straw biochar NPs, with no significant reduction in Na transport [153]. Rice straw nanobiochar’s ability to decrease Na^+^ translocation demonstrates a unique mechanism of biochar nanoparticles in salt stress alleviation. Zhang et al. [154] reported an increase in fruit yield (115%) and fruit quality with a 2% biochar in NP treatment compared to salt stress alone. Biochar NPs alleviate salt stress by regulating ion balance: limiting Na^+^ movement to leaves and fruits (reducing Na content (35%) in leaves and in fruits (16%)), enhancing K^+^ levels and Na^+^/K^+^ ratios, and affecting Mg and P absorption and translocation [154]. Therefore, alleviation of salt stress by biochar-based NPs involves regulation of ion homeostasis, limitation of Na^+^ translocation (leaves and fruits), improvement of the Na^+^/K^+^ ratio and K^+^ content, and subsequent absorption and translocation of Mg and P.

Recently, EDTA-chelated biochar and AMF showed high plant growth and physiology [155]. Cheng et al. [156] reported that *Serratia nematodiphila* biochar-based seed coating in *Zea mays* alleviates salt stress. Biochar and nanobiochar are gaining momentum in augmenting salt resilience and reducing GHG emissions. Enhanced ionic adsorption capabilities, nutrient availability, and soil properties aid in microbial benefits for nutrient cycling [157]. Biochar-based nanocomposites improve soil fertility, nutrient acquisition, and plant growth [158,159]. Altered physicochemical properties and Na+ absorption contribute to elevated nutrient uptake rates in saline soil, thereby enhancing stress tolerance [158]. In another study using biochar-based nanocomposites, they mitigate salt stress and increase the antioxidant properties of essential oil (monoterpenes) in dill (*Anethum graveolens*) seeds [160]. Positive attributes are fostered by nanosized biochar, its increased surface area, and its high reactivity. Foliar spray of nano biochar colloidal solution showed an increase in primary and secondary metabolites and antioxidant enzymes; improved growth and yield, pigment production, leaf water content, electrolyte leakage, and lipid peroxidation were also correlated with escalated tomato productivity [161]. The nanostructure of NPs and nanobiochar contributes to reduced evapotranspiration and increased soil aeration, thereby enhancing water-holding capacity. Dilution of excess Na^+^ ions increased mineral content and release of K^+^, Ca^2+^, and Mg^2+^ [159]. Similarly, the protective effects of nanobiochar were observed in enhanced salt tolerance in tomato, assuaging food security and resilient systems for sustainable agriculture that support the circular economy [161]. Nanobiochar’s effectiveness against salinity stress aligns with sustainable agriculture, emphasizing its enhanced physicochemical properties, including a large specific surface area and strong cation exchange capacity [161]. Nanobiochar-based organic fertilizers alleviated saline–alkaline soil stress [162]. Nonetheless, the utility of biochar-based biofertilizers vs. chemical fertilizers remains an under-explained research area. Hence, future directions and copious authentic research are needed to clarify the concept of agricultural sustainability. Table 1 lists the key mechanisms involved in salt stress alleviation by nanotechnology-based biochar and the corresponding experimental outcomes. However, long-term effects in climate-resilient crops and sustainable agriculture require additional prospective research. In the future, soil amendment with nanoscale biochar may be a highly effective tool for sustainably increasing food production and promoting global food security.

### 4.2. Status of Modified Biochar Against Salt Stress

Biochar is most commonly used for soil bioremediation due to its porous and aromatic structure, which offers a large surface area with different functional groups and high cation exchange capacity. Modifying and fortifying biochar is an important technique for the production of a variety of biochars with improved characteristics compared to those of mother biochar for stress amelioration and agricultural production. Chitosan-modified biochar, used against salt stress, can be prepared by chemical modifications, nanomaterial incorporation, and chitosan modifications. The action of chitosan-modified biochar on salt stress tolerance in soybeans showed multiple interactions. Wu et al. [163] produced magnesium oxide (MgO)–biochar to improve inorganic phosphate (Pi) adsorption in saline soils. MgO maximum Pi adsorption capacity was 1.46 times higher than Biochar. They also found that electrostatic attraction, precipitation, and exchangeable anions contributed to the adsorption of phosphate, and MgO–biochar possessed the greatest adsorption capacity. MgO–biochar application increased soil available P content and resulted in higher rice yields in field experiments under saline soil conditions. Salt stress amelioration mechanisms by modified biochar increase soil characteristics, nutrient availability, soil fertility, and altered soil microbial community dynamics [24,164,165]. Biological activity of modified biochars is confronted with pH modifications, enhanced plant growth, bacterial aggregations, nutrient uptake, and ROS enzymes [166,167,168].

Biochar-supplemented gypsum, lime, and farm manure synergistically induced salt stress tolerance in rice [169]. Silica-modified biochar reduced soil salinity, drought stress, and nutrient uptake in Safflower [170]. Soil improvement in saline–alkali soils relies on modifying appropriate functional groups and their effects on soil properties (pH, nutrients, and microbial activity) [171]. Hence, biochar modification methods and critical effects in synergizing salt stress mitigation and soil reclamation need to be revisited. Acid modifications of biochar (nitric acid) alter soil properties and biochemical nature (in saline–sodic soils [172]. Acid-modified biochar enhanced the growth and quality of spinach in coastal saline soils, compared to common biochar [173]. Iron-enriched biochar showed increased Na^+^ adsorption, enhanced soil health, and improved crop physiology under salt stress [174]. Biochar-based rhizobacteria (*Pseudomonas putida* RS-198 and *Azotobacter chrococcum* RS-106) revealed a novel strategy of nutrient uptake and salt stress tolerance in rapeseed [96]. Biochar integrated with *T. harzianum* promoted antioxidant activity, decreased ROS activity, and membrane leakage in Spinach [173]. Further, de-ashed biochar enhanced fenugreek cultivation under salinity stress [175]. Manure–biochar compost alleviated soil salinity by increasing osmosis, antioxidant activity, nutrient uptake, and photosynthetic efficiency and by reducing ROS and leaf damage in tomato plants [176].

Table 2 summarizes recent advances in modified biochar for mitigating salt stress and promoting soil reclamation. Overall, biochar, modified biochar, and nanotechnology-based biochar ameliorate salt stress in both soil and plants [177]. Thus, it is evident that soil-microbiota-modified biochar will enhance crop productivity in saline–sodic soils. Figure 2 depicts the future holistic management of biochar, modified biochar, and nanotechnology-based biochar against salt stress and soil reclamation. Hence, the intrinsic mechanisms deciphered address novel combinations for rationalizing salt stress alleviation by biochar, even in desert agricultural sustainability. Moreover, agglomerating the plant–soil–biochar nexus for all crop plants and abiotic stress management require authentic field trials, transitioning to large-scale outcomes envisaged by multi-omics interactions.

## 5. Bottlenecks in Biochar Application

Biochar-mediated salt stress alleviation presents challenges, including concerns about cost-effectiveness and limited data on long-term benefits. Additionally, the climate-related limitations for biochar remain complex. Region specificity and varied soil parameters also add to the complexity of plant growth and salt stress. The hydrophobicity of biochar, influenced by raw material and pyrolysis conditions, affects water retention. Hence, enhanced properties, such as biochar-based organo-fertilizers or nanobiochar, are stressed for a multitude of benefits. Further, the rise of nano pollution in climate resilience and global warming will provide future thrust. Emphasis is also placed upon unusual increase in soil salinity and sodicity due to biochar use. The roles of phytohormone signaling and their interplay need to be deciphered to understand complex crosstalk mechanisms in the biochar–plant–soil nexus. Soil health, focusing on soil microbiota, requires intricate assessments of community structure and ecosystem dynamics. Moreover, soil fertility and nutrient cycling are required for continuous monitoring and upregulation. Thus, fewer limitations upon direct attention pose significant sustainable outcomes.

## 6. Conclusions

The congregation effects of biochar on salt stress alleviation revealed altered physicochemical properties (water-holding capacity and enhanced antioxidant defense). Biochar production correlated with the use of various feedstocks, soil properties, plant type, and subsequent outcomes; the physical, chemical, and biological properties of biochar were associated with CEC, organic matter, and electrical conductivity. The biochar–plant–soil nexus interaction in deriving a holistic mechanism affirmed physicochemical and biological mechanisms. Further elucidation of phytohormone signaling mechanisms for salt stress amelioration provided a comprehensive understanding of the enhanced benefits of salt stress tolerance. Microbial community dynamics in soil health, homeostasis, and fertility were corroborated by the use of biochar-based fertilizers and nanotechnology-modified biochar. Future research on nanobiochar for environmental impacts will focus on reducing GHG emissions and on climate-resilient crops. Further research on biocompatibility, biogeochemical cycling, phytohormonal crosstalk, and signaling interplay is needed for effective and sustainable agriculture. The present review also ascertains the future imperatives of biochar-enhanced salt stress alleviation in achieving sustainable development goals.

## Figures and Tables

**Table 1:** Nanotechnology-modified biochar effect on salinity stress alleviation.

Nanobiochar	Underlying Mechanism	References
Biochar and Selenium (Se)-chitosan nanoparticles in Soybean	Cellular injury protection, ionic homeostasis and carbon assimilation, upregulation of key transporter genes	[90]
Nanocollodial solution for tomato	Improved crop nutritional status under salinity stress	[161]
Biochar-based nanocomposites on dill (*Anethum graveolens*)	Increased iron and zinc content and decreased Na+ accumulation in leaf. Increased production of monoterpenes and antioxidant activity	[158]
Biochar-loaded biofertilizers, nano-zeolite and nano-silicon in *Thymus vulgaris*	Soil salinity amelioration, increased plant growth and productivity	[87]
Chitosan modified biochar in soybean	Enhanced soil plant interactions, nutrient uptake augmented	[101]
Biochar-based nanocomposites in plants	Enhanced salt tolerance, plant biomass and essential oil through low Na^+^ content and ROS generation, increased nutrient availability, water status and photosynthetic pigments	[93]
Iron and zinc-modified nanobiochar in rice	Nutrient availability, microbial community, salinity Stress and Food Security	[145]
Nano biochar on the growth and productivity of tomato plants	Increased yield and physiological characteristics of plants	[153]
Biochar nanoparticles alleviate salt stress in tomato plants	Reduced the accumulation of Na^+^ content in plants and increased biomass and chlorophyll content	[154]

**Table 2:** Recent advances in modified biochar regarding effects against salt stress.

Nature of Modified Biochar	Mechanism	References
Organic amendment with *Gliricidia sepium* biochar at 500 °C	Increased physico-biochemical soil properties	[178]
Combined effect of Rice straw biochar and humic acid	Enhanced nutrient antioxidant activity and ion uptake in Maize	[179]
CaSO_4_·2H_2_O in Titanium-gypsum biochar	Improved plant growth against salinity stress	[180]
Phosphorous-Mg modified biochar included PA-Mg and DAP-Mg biochar from *Spartina alterniflora*	Salinity stress mitigation enhanced bacterial community structure and plant growth in *Suaeda salsa*	[181]
Acid modified biochar	Increased yield and quality of Spinach in saline soils along coasts	[173]
Biochar amended with PGPR (*Azotobacter chrococcum* and *Pseudomona koreensis*	Enhanced plant growth of maize in salt affected soil and saline–sodic soil	[182]
Biochar and polyacrylamide	Improved rice growth and enhanced coastal saline soil quality	[183]
Nitric acid modified biochar	Enhanced organic matter, nitrogen and available phosphorous and potassium. Improved SOD, POD, CAT and reduced MDA and H_2_O_2_	[184]
Biochar with glycine betaine	Improved yield against salinity	[185]
Calcium modified biochar	Phytoremediation (*Thinopyrum ponticum*) of human induced salt pollutants	[186]
Hardwood modified biochar	Increased phosphorous availability and uptake in saline alkali soil	[187]
Residual sulfur enhanced biochar	Increased N, P, K^+^, Ca^2+^, Fe, Mn, Cu, and Zn uptake in Capsicum annum	[188]
Calcium modified biochar	Enhanced soil properties of saline–alkali soil	[189]
AMF and biochar	Improved salinity tolerance, plant growth and lipid metabolism in Wheat	[190]
Biochar-manure compost with Pyroligneous solution	Salt stress alleviation and yield of wheat in stressed crop land	[191]
Nanobiochar foliar spray	Alleviated salt stress and enhanced growth in tomato	[184]
Synergistic biochar Silicon	Enhanced salt tolerance, microbiome shifts and nutrient cycling	[192]

## Data Availability

The original contributions presented in this study are included in the article. Further inquiries can be directed to the corresponding author.
